# Nickel Carcinogenesis Mechanism: DNA Damage

**DOI:** 10.3390/ijms20194690

**Published:** 2019-09-21

**Authors:** Hongrui Guo, Huan Liu, Hongbin Wu, Hengmin Cui, Jing Fang, Zhicai Zuo, Junliang Deng, Yinglun Li, Xun Wang, Ling Zhao

**Affiliations:** 1 College of Veterinary Medicine, Sichuan Agricultural University, Wenjiang, Chengdu 611130, China; guohonrui@163.com (H.G.); lhuansy@163.com (H.L.); hongjun1ca@126.com (H.W.); fangjing4109@163.com (J.F.); zzcjl@126.com (Z.Z.); dengjl213@126.com (J.D.); liyinglun01@163.com (Y.L.); wangxun99@163.com (X.W.); lingzhao@sicau.edu.cn (L.Z.); 2 Key Laboratory of Animal Diseases and Environmental Hazards of Sichuan Province, Sichuan Agriculture University, Wenjiang, Chengdu 611130, China; 3 Key Laboratory of Agricultural information engineering of Sichuan Province, Sichuan Agriculture University, Yaan 625014, Sichuan, China

**Keywords:** Ni, carcinogenicity, DNA damage, ROS, DNA damage repair

## Abstract

Nickel (Ni) is known to be a major carcinogenic heavy metal. Occupational and environmental exposure to Ni has been implicated in human lung and nasal cancers. Currently, the molecular mechanisms of Ni carcinogenicity remain unclear, but studies have shown that Ni-caused DNA damage is an important carcinogenic mechanism. Therefore, we conducted a literature search of DNA damage associated with Ni exposure and summarized known Ni-caused DNA damage effects. In vitro and vivo studies demonstrated that Ni can induce DNA damage through direct DNA binding and reactive oxygen species (ROS) stimulation. Ni can also repress the DNA damage repair systems, including direct reversal, nucleotide repair (NER), base excision repair (BER), mismatch repair (MMR), homologous-recombination repair (HR), and nonhomologous end-joining (NHEJ) repair pathways. The repression of DNA repair is through direct enzyme inhibition and the downregulation of DNA repair molecule expression. Up to now, the exact mechanisms of DNA damage caused by Ni and Ni compounds remain unclear. Revealing the mechanisms of DNA damage from Ni exposure may contribute to the development of preventive strategies in Ni carcinogenicity.

## 1. Introduction

Nickel (Ni) is the 24 th most abundant element in Earth’s crust [1]. On the one hand, at a low concentration, Ni is a nutritionally essential trace element for animals [2,3,4]. Ni is also a required trace element for several enzymes that play critical roles in energy and nitrogen metabolism [5]. On the other hand, at a high concentration, Ni is a toxic element [6,7,8,9]. The unique physical and chemical properties of Ni make it and its compounds suitable materials for many applications widely found in modern industries [1]. The widespread use of Ni increases its concentration in biogeochemical cycles and enhances human exposure to it and its compounds through environmental contamination and occupational exposure [10]. Human exposure to Ni occurs primarily via inhalation, ingestion, and dermal absorption [11]. In humans, Ni has been known to cause liver, kidney, spleen, brain, and tissue damage [12,13,14]. The International Agency for Research on Cancer (IARC) has classified Ni compounds such as nickel sulfate (NiSO_4_), nickel oxide (NiO), nickel hydroxides, and crystalline nickel as carcinogenic agents to humans (Group 1) [15].

It has been confirmed in many in vitro and vivo studies that Ni and Ni compounds have carcinogenicity [16,17,18,19,20,21,22]. Epidemiological studies presented that the probability of lung and nasal cancers are significantly increased in nickel-exposed workers [6]. Up to now, the exact mechanism of Ni carcinogenicity is still unclear, but it is clear that DNA damage is an important part of it [21,23,24,25]. After DNA damage occurs, cells activate several response signals, such as cell-cycle arrest, DNA repair, and cell death [26,27,28]. Numbers in in vitro and in vivo studies have presented that Ni and Ni compounds can induce DNA damage [29,30,31,32,33]. We also found that DNA oxidative damage and cell cycle arrest can be induced by dietary nickel chloride (NiCl_2_) in excess of 300 mg/kg in the thymus, the bursa of Fabricius, the kidney, and the liver of broiler chickens [34,35,36,37]. If the DNA repair system cannot repair the damaged DNA, and the damaged DNA is passed onto the daughter DNA, this causes genomic instability, which finally promotes cancer development [38,39,40,41]. In this review, we paid attention to the effects of DNA damage and DNA repair system inhibition induced by Ni.

## 2. Ni-Induced DNA Damage

In general, several chemicals, radiation, and free radicals can affect the DNA and induce DNA damage [42]. The process of carcinogenesis is always initiated by the DNA damage [43], and damaged DNA replication may lead to gene mutation, which in turn induces protein alteration and cancer development [39,44,45,46].

In humans, DNA damage is very serious among Ni-smelting workers [47]. Previous studies found that Ni^2+^ can also induce DNA damage in various human cell systems, including human hepatocellular carcinoma (HepG2) [48], human TK6 [49], Chinese hamster lung fibroblast [50], A375 [51] and HCT-116 cells [52]. When human B lymphoblastoid cells were exposed to NiCl_2_ (0.63mM) for 24 and 48 h, cellular DNA damage was significantly increased when compared to the control cells [53]. A comet assay and γ-H2AX immunofluorescence staining showed that nickel acetate-induced significant DNA damage in human colon-cancer cells (RKO) [54].

Animal studies have also shown that Ni or Ni compounds can cause DNA damage. In rats, comet-assay studies showed that single-strand breaks were observed in rat lungs and kidneys after acute treatment of animals with injected NiCl_2_ (44.4 mg/kg body weight), and the lung was the most susceptible tissue to NiCl_2_ [55]. Our studies have also indicated that dietary NiCl_2_ in excess of 300 mg/kg can induce DNA damage in the lung and the kidney of broiler chickens [56,57]. In addition, overexposure of nickel nitrate [Ni(NO_3_)_2_] can induce DNA damage in earthworms (*Eisenia foetida*) [58], and NiCl_2_ can induce DNA damage in *Caenorhabditis elegans* [59].

## 3. Binding of Ni to DNA and Nuclear Proteins in Ni-Induced DNA Damage

Previous data have demonstrated that a portion of Ni ions can enter the nucleus after exposure to Ni and Ni compounds [60,61]. Fletcher et al. [62] found that exposure of cells to water-soluble Ni salts resulted in very low nuclear but high cytosolic Ni levels, while exposure to insoluble Ni salts caused relatively high nuclear levels. It has also been confirmed that Ni ions exhibit a lower binding affinity for DNA, thus most nickel ions in the cell nucleus could interact with the histone [63,64,65,66,67]. Oliveria et al. [68] clearly demonstrated that Ni^2+^ interacts by binding to dsDNA strands causing conformational changes. The interaction of DNA with Ni has been extensively investigated since they are involved in processes leading to DNA damage [68]. Ciccarelli et al. [69] demonstrated the presence of Ni-nucleic acid histone complexes in Ni-treated rats and suggested that Ni may initiate DNA damage by forming this complex. Binding of Ni to chromatin DNA and associated proteins has been reported to cause DNA damage, which consists of DNA single-strand breaks and DNA intrastrand cross-linking [70,71]. In recent decades, coordination compounds with Ni have become quite important in medicinal chemistry, and their research data show that Ni(II) complexes can wind DNA strands through groove interactions and promote strand breakage [71].

## 4. Reactive Oxygen Species (ROS) in Ni-Induced DNA Damage

**Figure 1:**
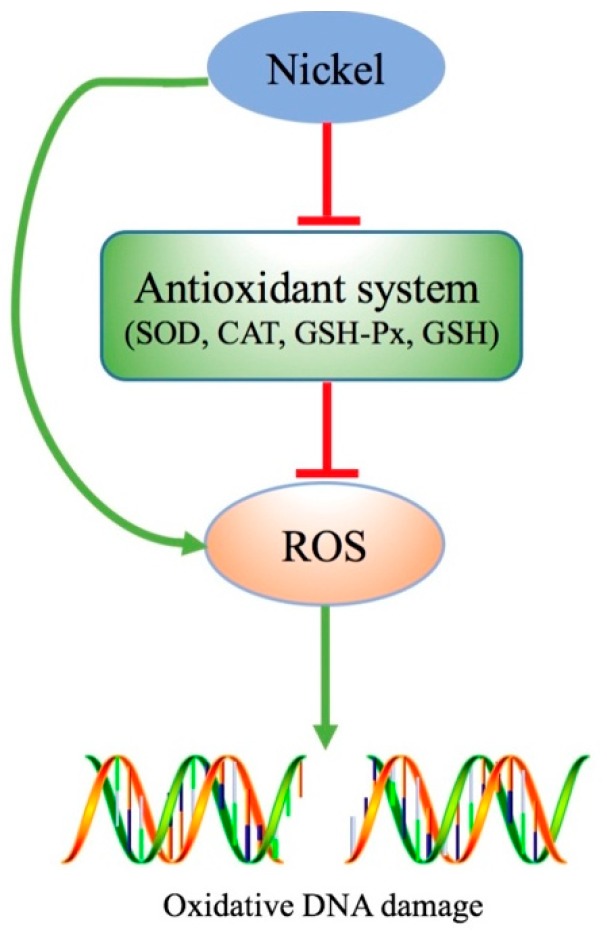
Ni induced reactive oxygen species (ROS) accumulation. Excessive exposure of Ni can increase ROS accumulation through directly increasing ROS generation and through an antioxidant system suppression, which then damages the DNA.

ROS are a group of short-lived, highly reactive, oxygen-containing molecules [72]. ROS play an important role in cancer development [73]. Excessive ROS attack the DNA, which then results in genomic instability that is a promoter of tumorigenesis [72]. Genomic instability has been suggested to be a major driving force of oncogenesis and can account for genetic diversity in many cancers [72]. It has been shown that oxidative stress is the basic toxicological mechanism of Ni overexposure [33,51,74,75]. Ni and Ni compounds increase ROS accumulation through both a direct increase in ROS generation and an antioxidant-system suppression, which then damages the DNA (as shown in Figure 1).

### 4.1. Ni-Induced ROS Accumulation

Numerous in vitro and in vivo studies have shown that Ni and Ni compounds can induce ROS accumulation and oxidative stress [76,77,78,79,80,81,82,83]. Ni and Ni compounds induce ROS accumulation in two ways: (i) increasing ROS generation and (ii) impairing the antioxidant system [84,85]. Our previous data indicated that dietary NiCl_2_ in excess of 300 mg/kg can suppress the activities of antioxidant enzymes such as superoxide dismutase (SOD), catalase (CAT), and glutathione peroxidase (GSH-Px), and glutathione (GSH) contents in kidney, lung, thymus, spleen, bursa of Fabricius, intestine, and cecal tonsil [56,57,86,87,88,89,90]. Ni nanoparticles (NiNPs) (45 mg/kg) treatment for 10 weeks increased ROS generation and decreased SOD, CAT activities, and GSH contents in rat testes [76]. Ahamed et al. also reported that culturing with 25–100 μg/mL nickel oxide nanoparticles (NiONPs) for 24 h can promote ROS accumulation in human hepatocytes (HepG2), and NiONPs caused cytotoxicity mainly via ROS [91]. ROS play a critical role in Ni-induced apoptosis and DNA damage [92,93,94,95,96]. Ni exposure can generate ROS in exposed cells, and ROS generation mediates biological effects in nickel-treated cells, which may play a role in nickel-induced carcinogenesis. ROS production is a critical factor in Ni toxicity and is also an indispensable element in Ni carcinogenesis [97,98,99].

### 4.2. ROS-Dependent Ni-Induced DNA Damage

Excessive ROS can directly attack the DNA by oxidizing nucleoside bases, producing modified nucleotides (8-hydroxy-2′-deoxyguanosine, 8-OHdG) [100,101,102]. The elevated 8-OHdG level is regarded as an indicator of DNA oxidative damage [103]. It has been suggested that synergistic DNA damage induced by simultaneous exposure of Ni compounds is possibly related to ROS [53,104,105]. Several in vitro and in vivo studies have demonstrated that Ni and Ni compounds can increase DNA oxidative damage marker levels (8-OHdG) [106,107,108,109,110,111]. In vivo, our previous studies showed that dietary NiCl_2_ in excess of 300 mg/kg inhibits the antioxidant system, which leads to an increase in DNA oxidative damage markers, e.g., 8-OHdG contents in the lung and the kidney [56,57]. Liu et al. found that treatment with NiSO_4_ (20 mg/kg) for 20 days induced ROS accumulation and increased 8-OHdG levels in mouse livers [107]. Treatment of cultured HeLa cells with Ni_3_S_2_ (10 µg/mL) can largely increase 8-OHdG contents, whereas NiO (black), NiO (green), and NiSO_4_ do not enhance the production of 8-OHdG [111]. NiCl_2_ (0.125, 0.25, and 0.5 mM) treatment for 24 h can also induce mitochondrial DNA (mtDNA) damage, including increased mitochondrial 8-OHdG contents and reduced mtDNA contents and mtDNA transcript levels in Neuro2a cells [112]. In recent decades, several studies have reported that Ni and Ni compounds nanoparticles of can also induce DNA damage [113,114,115,116,117,118]. Mo et al. [113] reported that oxidative DNA damage was significantly upregulated in the lungs of mice after being intratracheally instilled with 50 µg Nano-Ni. In vitro, Abudayyak et al. reported that NiO NPs induced excessive ROS generation and then caused DNA oxidative damage (8-OHdG up-regulation) in NRK-52E kidney epithelial cells and SH-SY5Y neuronal cells [114,115]. It has been also shown that Ni and NiO NPs at higher doses (25 and 50 ug/mL) can induce DNA strand breaks, and an increase in the DNA strand breaks is due to intracellular ROS generation [119].

Through co-treatment with the antioxidant N-acetylcysteine (NAC) and NiNPs, NAC can mitigate NiNPs-induced ROS generation and DNA strand breaks, suggesting the potential mechanism of ROS in DNA damage [120]. Ni acetate can increase intracellular ROS generation and DNA strand breaks in Nrf2 knockdown cells, indicating that ROS play an important role in Ni-induced DNA damage [54].

### 4.3. ROS-Independent Ni-Induced DNA Damage

In contrast to most studies, Kumar et al. demonstrated that NiSO_4_ affected DNA replication and damaged DNA but did not induce any detectable ROS production in *Escherichia coli*, concluding that Ni-induced DNA damage is through an ROS-independent pathway [121]. They also demonstrated that Ni exposure specifically affected DNA polymerization and thereby induced DNA damage. A major reason for the differing results may be related to differences between mammals and bacteria.

## 5. Interference of Ni with DNA Damage Repair Systems

After DNA damage occurs, specific pathways are activated to facilitate the identification of the damaged regions and their repair [122]. The DNA repair system contributes to the maintenance of the genetic sequence, the correction of DNA damage, and genomic instability [123,124].

Some studies showed that Ni can inhibit the DNA repair function and promote carcinogenesis [121,125,126,127,128,129]. Arita et al. reported that 29 DNA repair genes were repressed, and two DNA repair genes were overexpressed in the isolated peripheral blood mononuclear cells (PBMC) in Ni-refinery workers [130]. In addition, Scalon et al. [129] reported that DNA double-strand breaks were significantly higher in the NiCl_2_ (250 and 500 μM, 48 h) treatment cells than non-NiCl_2_ treatment cells after ionizing radiation (IR) exposure. Likewise, Ni also causes DNA damage repair-system repression, which results in damaged DNA not being removed. The accumulation of DNA lesions facilitates the process of tumorigenesis [131]. At present, an increasing number of studies indicate that DNA repair suppression is a non-ignorable mechanism of oncogenesis [43,132,133].

**Figure 2:**
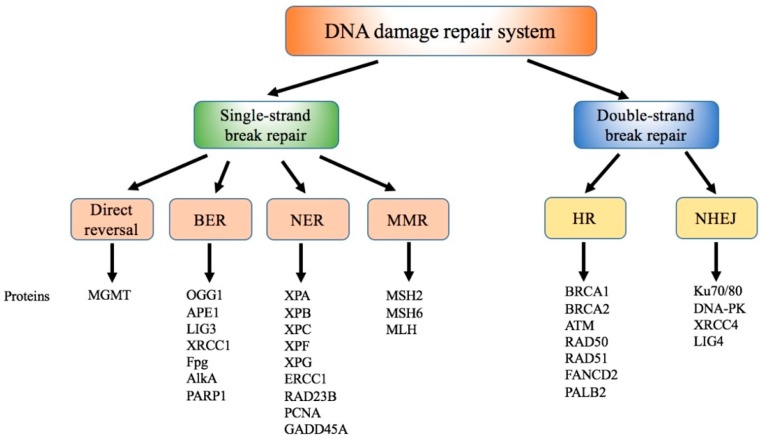
DNA damage repair systems. DNA damage repair systems include direct reversal, base-excision repair (BER), nucleotide repair (NER), mismatch repair (MMR), homologous-recombination repair (HR), and nonhomologous end joining (NHEJ) repair pathways.

In general, the DNA repair system can always fix and clear damaged DNA that is induced by normal metabolic activities and environmental factors [134]. Recently, the DNA repair mechanism was found to contain direct reversal, nucleotide repair (NER), base excision repair (BER), mismatch repair (MMR), and double strand break repair including homologous recombination repair (HR) and nonhomologous end joining (NHEJ) repair [135,136], as shown in Figure 2. Next, we review the effect of Ni on every DNA damage-repair pathway (Table 1).

### 5.1. Effect of Ni on Direct Reversal

For reversible DNA damage, our body first uses the direct-reversal repair mechanism to correct damaged bases [149]. The basic means of DNA repair way is direct reversal, which corrects damaged DNA with DNA alkylating agents [149]. O^6^-methylguanine DNA methyltransferase (MGMT) and ALKBH α-ketoglutarate Fe(II) dioxygenases (FeKGDs) are the main direct reversal repair proteins [150].

In nickel sulfide (NiS)-treated or NiS-transformed human 16HBE cells, the DNA repair gene MGMT mRNA and protein-expression levels are significantly reduced, suggesting that the downregulation of MGMT expression levels may be an early event involved in NiS-induced cell transformation [137]. MGMT is unique among DNA repair proteins because it acts alone to remove DNA adducts [151]. MGMT repair can remove both methyl and ethyl adducts, and this reaction is a nonenzymatic (stoichiometric) reaction. Previous studies confirmed that MGMT overexpression can increase the resistance to cancer. MGMT silencing is also associated with DNA hypermethylation, histone modifications, and DNA methyltransferase 1 (DNMT1) upregulation [137]. However, Iwitzki et al. found that Ni treatment does not affect the MGMT protein levels and only inhibits the MGMT activity in Chinese hamster ovary cells (CHO) cells [138]. These differences are possibly due to the different cell types under study or the differences in duration of Ni exposure. 

The other type of direct-reversal repair is performed by ALKBH proteins that are members of a superfamily of FeKGDs [152]. Only four ALKBH family proteins (ALKBH1-3 and FTO) have the ability of DNA alkyl damage removal [153]. Ni can also directly inhibit the DNA alkylation repair enzymes, e.g., ALKBH2 and ALKBH3, by replacing iron at the catalytic site and then reducing the direct reversal of DNA damage [139,140].

### 5.2. Effect of Ni on BER

BER mainly repairs oxidative DNA damage, and it can excise and replace a single damaged nucleotide base [154]. The process of BER has two steps. First, DNA glycosylase finds and cuts the damaged DNA base. Second, the DNA repair proteins repair the damaged site [154]. At least 11 distinct mammalian DNA glycosylases are known, such as 8-oxoguanine DNA glycosylase (OGG1), AP endonuclease (APE1), DNA ligase 1 (LIG1), LIG3, and X-ray repair cross-complementing protein 1 (XRCC1) [122,155].

In a nickel smelting worker’s serum, the oxidative DNA damage marker (8-OHdG) levels are significantly increased, and BER DNA glycosylase human OGG1 (hOGG1) is significantly lower than that of nonexposed workers [47]. hOGG1 is a single BER enzyme, specifically recognizing and repairing DNA oxidative damage by removing 7,8-dihydro-8-oxoguanine (8-oxoG). Downregulation of hOGG1 expression is associated with aging, neurodegenerative disorders, and cancer [155,156]. Additionally, Ni at 1 μM can inhibit the activity of formamidopyrimidine-DNA glycosylase (Fpg) and 3-methyladenine-DNA glycosylase II (Alk A), which are involved in DNA excision repair [141]. Fpg is a glycosylase-initiating BER enzyme in *E. coli* and participates in the first step of the BER to remove specific modified bases from the DNA.

### 5.3. Effect of Ni on NER

The aim of NER is to repair DNA damage that has two or more base-impair sites [157,158]. The process of NER entails damaged-site recognition, damaged-strand removal, and DNA ligation [159,160,161,162]. The NER process needs the participation of many proteins, such as Xeroderma pigmentosum (XP) complementation Groups A through G, excision repair cross-complementation group 1 (ERCC1), and proliferating cell nuclear antigen (PCNA) [163,164].

The NER pathway plays an important role in the prevention of cancer formation, such as bladder cancer and lung cancer [165]. It has been shown that Ni inhibits the removal of UV-induced DNA damage by disturbing DNA repair proteins and affecting the NER process [127,142,143,144]. Hartwig et al. showed that Ni inhibits NER through the disruption of the incision step in HepG2 and CHO cells [127,145]. The preferential binding of Ni(II) to the protein (DNA repair enzyme) fraction, as compared to DNA and RNA fractions, is the reason for NER incision-step inhibition [146]. Ni also inhibits the NER incision step, possibly through direct interaction with zinc finger repair enzymes such as XPA and poly ADP-ribose polymerase (PARP), and the displacement of the zinc ions [125,126,127,128,131,144,166].

Kim et al. [147] found that treatment with nickel acetate 20 μM for 24 h can induce p53-mediated NER DNA repair-pathway suppression, which is a promoter of tumor development. Nickel acetate inhibits p53 transcriptional activity and then suppresses DNA-damage-inducible protein 45 alpha (GADD45A) expression. Typically, the GADD45A, PCNA, and XPG complex works in the NER incision process, and the function of this complex is site 3′ of damage cleavage [167]. Downregulation of GADD45A expression levels results in GADD45A, PCNA, and XPG complex inhibition, which then impairs the GADD45A-mediated NER mechanism [147].

### 5.4. Effect of Ni on MMR

DNA MMR is also a critical pathway for DNA damage. The MMR system maintains genomic stability by repairing base–base mismatches and insertion/deletion loops that arise from DNA replication, thereby preventing mutations from becoming permanent in dividing cells [168,169]. Defects in MMR increase the spontaneous mutation rate and sporadic human cancers [170,171]. MMR is a complex reaction involving multiple proteins that recognize a mismatched base, excise the DNA damage, and resynthesize the DNA sequence containing the correct base and using the parental strand as a template [172]. In general, MMR is initiated when highly conserved proteins (MutS homolog 2 MSH2 and MSH6) recognize single base mismatches [173]. In cases of insertion/deletions loops with two or more extra bases, MSH2 and MSH3 are responsible for detection. Following recognition, one of the mutL homolog (MLH) heterodimers binds to the mismatch, and PCNA is loaded onto the DNA by replicating factor C, activating MLH to incise the nascent strand and removing the error in an ATP-dependent manner [174,175]. Then, DNA polymerase synthetizes the new strand followed by nick-sealing [176,177].

Scanlon et al. found that treatment with NiCl_2_ 250 and 500 μM for 48 h could decrease MMR MLH1 protein and mRNA expression levels in tumorigenic (A549) and nontumorigenic (BEAS-2B) human lung cells [129], and that NiCl_2_ could directly bind to the MLH1 gene promoter and then reduce its activity of it. However, it has been demonstrated that there are no alterations of DNA repair genes including hMLH1 and hMSH6 in NiS-transformed 16HBE cells [137].

### 5.5. Effect of Ni on Double-Strand Breaks Repair Pathways

Double-strand breaks (DSB), a DNA damage type, causes changes to the DNA sequence [178]. After DSB occur, repair systems NHEJ or HR are activated [179]. HR, a conservative process, fixes the damaged DNA to be the same as the original DNA sequence. In this process, the damaged DNA sequence is removed, and the new DNA synthesis is according to the homologous sister chromatid [180]. The proteins involved in the HR are shown in Figure 2, including breast cancer 1 (BRCA1), BRCA2, RAD51 (human homolog of *Saccharomyces cerevisiae* RAD50), fanconi anemia group D2 protein (FANCD2), and partner and localizer of BRCA2 (PALB2) genes [180,181].

In mammalian cells, NHEJ is a preferential way to repair DSB because a homologous template is not necessarily needed in NHEJ rejoins [182]. In NHEJ, the Ku70/Ku80 heterodimer recognizes and binds the two ends of the broken DNA strands [183]. Multiple enzymes are involved in the rejoining process, including LIG4, X-ray repair cross complementing 4 (XRCC4), and DNA-dependent protein kinase (DNA-PK) [183]. LIG4, XRCC4, and DNA-PK heterodimers are recruited by the Ku70/Ku80 heterodimer to the damage site promoting the ligation of the two ends [182].

Takahashi et al. [184] reported that treatment with 40 mM or more of NiCl_2_ for 30 min could inhibit the repair of DNA double-strand breaks in Chinese hamster ovary cells. NiCl_2_ also inhibited DNA repair only at cytotoxic concentrations at which the cells lost their proliferative ability. Scanlon et al. found that NiCl_2_ (250 and 500 μM for 48 h) exposure led to the downregulation of HR without downregulation of NHEJ repair, and that NiCl_2_ decreased the HR proteins (BRCA1, RAD51, and FANCD2) and the mRNA expression levels in tumorigenic (A549) and nontumorigenic (BEAS-2B) human lung cells [129]. There were no changes in the NHEJ repair proteins (DNA-PK, KU80, XRCC4, LIG4 protein, and mRNA expression levels) in tumorigenic (A549) and non-tumorigenic (BEAS-2B) human lung cells [129]. The mechanism of the NiCl_2_-inhibited HR pathway is through the transcriptional repression of DNA repair proteins. After evaluating the DSB DNA repair pathway by the ex vivo GFP reporter assay system, Morales et al. found that NiCl_2_ at low doses (100 μM) activated the HR pathway but did not affect the NHEJ repair pathway [148]. In contrast, the highest tested dose of NiCl_2_ (500 μM) significantly inhibited the HR and the NHEJ repair pathways in U2OS cells [148]. The different results of Morales et al. and Scanlon et al. on the NiCl_2_-affected NHEJ repair pathway may be because of NiCl_2_ treatment concentration or the cell model.

### 5.6. Others

When bacterial DNA encounters heavy doses of DNA-damaging agents, it activates the special DNA repair-system SOS response [185]. In general, SOS response repairs the damaged DNA and ensures the process of DNA replication [186]. If the SOS response is suppressed, the evolution of bacterial resistance and pathogens is prevented more easily [187]. Kumar et al. [121] reported that exposure to 1 M Ni ions for 15 h induced double-strand breaks of *E. coli*. In DNA, meanwhile, the SOS response is impaired by RecBCD function blockage.

## 6. Conclusions and Future Perspectives

**Figure 3:**
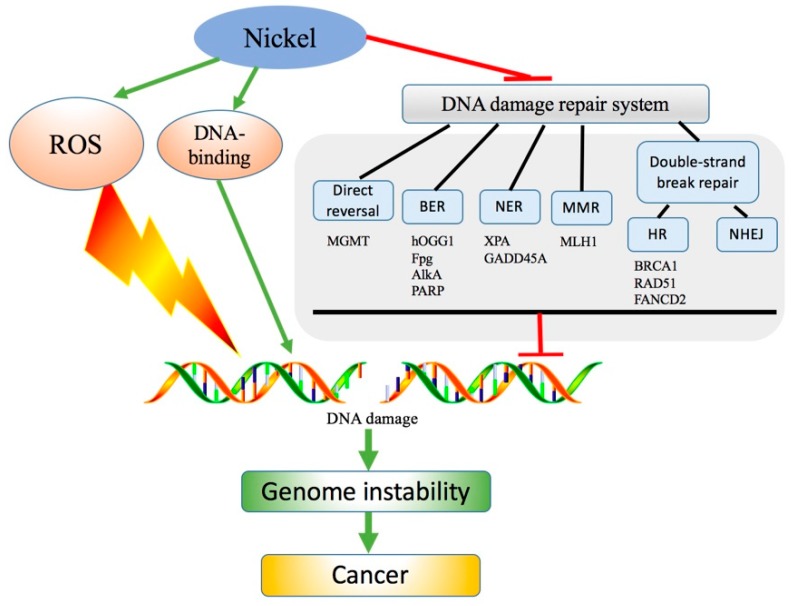
Simplified scheme of Ni-induced DNA damage in cancer occurrence. Excessive exposure to Ni can induce DNA damage, mainly through direct DNA binding and ROS generation. Ni can also repress the DNA damage-repair pathways, including direct reversal, BER, NER, MMR, HR, and NHEJ repair. DNA damage causes genome instability that may ultimately contribute to cancer occurrence.

There have been many studies on the molecular mechanism of Ni and Ni compounds-induced DNA damage associated with carcinogenesis. However, the exact mechanisms of DNA damage caused by Ni and Ni compounds are still unclear. Previous studies have demonstrated that Ni can induce DNA damage, and that Ni-induced DNA damage is mainly through ROS generation. Ni can also directly bind DNA and induce DNA damage. Meanwhile, Ni can also repress the DNA damage-repair systems, including DNA direct-reversal, NER, BER, HDR, MMR, and NHEJ repair pathways, which increases the accumulation of the damaged DNA bases. The repression of DNA repair is through impacting cellular DNA repair on multiple levels, from direct enzyme inhibition to the modulation of DNA repair-molecule expression (Figure 3). Ni exposure can therefore directly induce cancer through DNA damage and DNA damage-repair inhibition. On the other hand, DNA damage-repair inhibition induced by Ni can also increase the risk of other agents (ultraviolet light, ionizing radiation, chemicals, etc.) promoting caner. Ni-induced DNA repair pathway suppression results in damaged DNA accumulation in the cells. If the damaged DNA cell can survive, the damaged DNA is passed down through the damage site to daughter cells, which thus contributes to potential carcinogenesis.

An increasing number of studies confirm that ROS generation is a basis mechanism of Ni toxicity [6]. However, there are no studies about the ROS generation in Ni-induced DNA damage repair inhibition. In the next study, we need more research to explore whether ROS are also essential or just participates in Ni-induced DNA damage-repair pathway inhibition in the carcinogenesis.

## Figures and Tables

**Table 1:** Effect of Ni on DNA damage-repair systems.

Item	Suppression	Enhancement	No Alteration
Direct reversal	Ji et al. [137], Iwitzki et al. [138], Chen et al. [139], Chervona et al. [140]		
BER	Wu et al. [47], Wozniak and Blasiak [141]		
NER	Hartwig et al. [127], Hu et al. [142], Lee-Chen et al. [143], Wozniak et al. [144], [145], Hartmann and Hartwig [146], Hartwig et al. [128], Hu et al. [125], Wozniak and Blasiak [25], Kim et al. [147]		
MMR	Scanlon et al. [129]		Ji et al. [137]
HR	Scanlon et al. [129]		
NHEJ	Morales et al. [148]		Scanlon et al. [129]

## Abbreviations

ROS	reactive oxygen species
NER	nucleotide repair
BER	base excision repair
MMR	mismatch repair
HR	homologous recombination repair
NHEJ	non-homologous end joining
ER	endoplasmic reticulum
SOD	superoxide dismutase
CAT	catalase
GSH-Px	glutathione peroxidase
GSH	glutathione
8-OHdG	8-hydroxy-2′-deoxyguanosine
MNNG	N-methyl-N′-nitro-N-nitrosoguanidine
MNU	N-methyl-N-nitrosourea
MMS	methyl methanesulfonate
MGMT	O^6^-Methylguanine DNA methyltransferase
FeKGDs	ALKBH α-ketoglutarate Fe(II) dioxygenases
NiS	nickel sulfide
DNMT1	DNA methyltransferase 1
OGG1	8-oxoguanine DNA glycosylase
APE1	AP endonuclease
LIG1	DNA ligase 1
XRCC1	X-ray repair cross-complementing protein 1
Fpg	formamidopyrimidine-DNA glycosylase
AlkA	3-methyladenine-DNA glycosylase II
XP	xeroderma pigmentosum
ERCC1	excision repair cross-complementation group 1
PCNA	proliferating cell nuclear antigen
PARP	poly ADP-ribose polymerase
GADD45A	DNA-damage-inducible protein 45 alpha
MLH	mutL homolog
DSB	double-strand breaks
BRCA1	breast cancer 1
RAD51	human homolog of S. cerevisiae RAD50
f FANCD2	anconi anemia group D2 protein
PALB2	partner and localizer of BRCA2
XRCC4	X-ray repair cross complementing 4
DNA-PK	DNA-dependent protein kinase
CHO	Chinese hamster ovary cells
